# Supplementary material for: Monitoring training and recovery responses with heart rate measures during standardized warm-up in elite badminton players
Source: PLoS One. 2020 Dec 21;15(12):e0244412. doi: 10.1371/journal.pone.0244412 (PMC7751974; doi:10.1371/journal.pone.0244412)
Supplement: S1 Appendix — Individual monitoring data are visualized for each player during the observational period. (PDF) [file pone.0244412.s002.pdf]

Player A

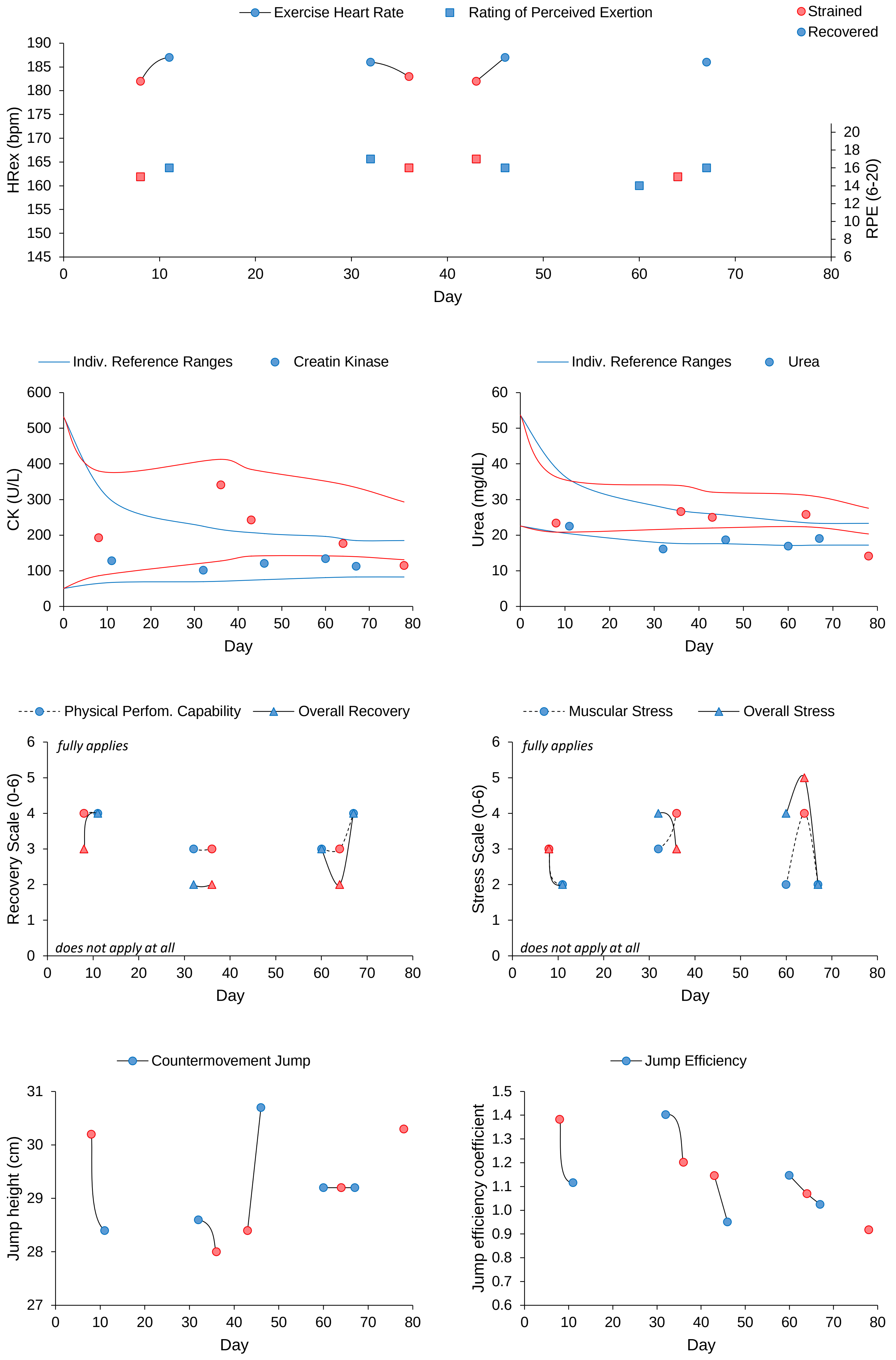

Individual data during the observational period. Blue dots: 'recovered' state (Mondays), red dots: 'strained' state (Fridays). Lines between data points are interrupted if measurements are not available or missing. HRex: exercise heart rate, RPE: rating of perceived exertion, CK: creatine kinase. Plots for CK and Urea also display individualized reference ranges for 'strained' (red lines) and 'recovered' data (blue lines) (95% confidence intervals), see Hecksteden et al. (2017) for reference and spreadsheets: https://doi.org/10.1123/ijspp.2016-0120

Player B

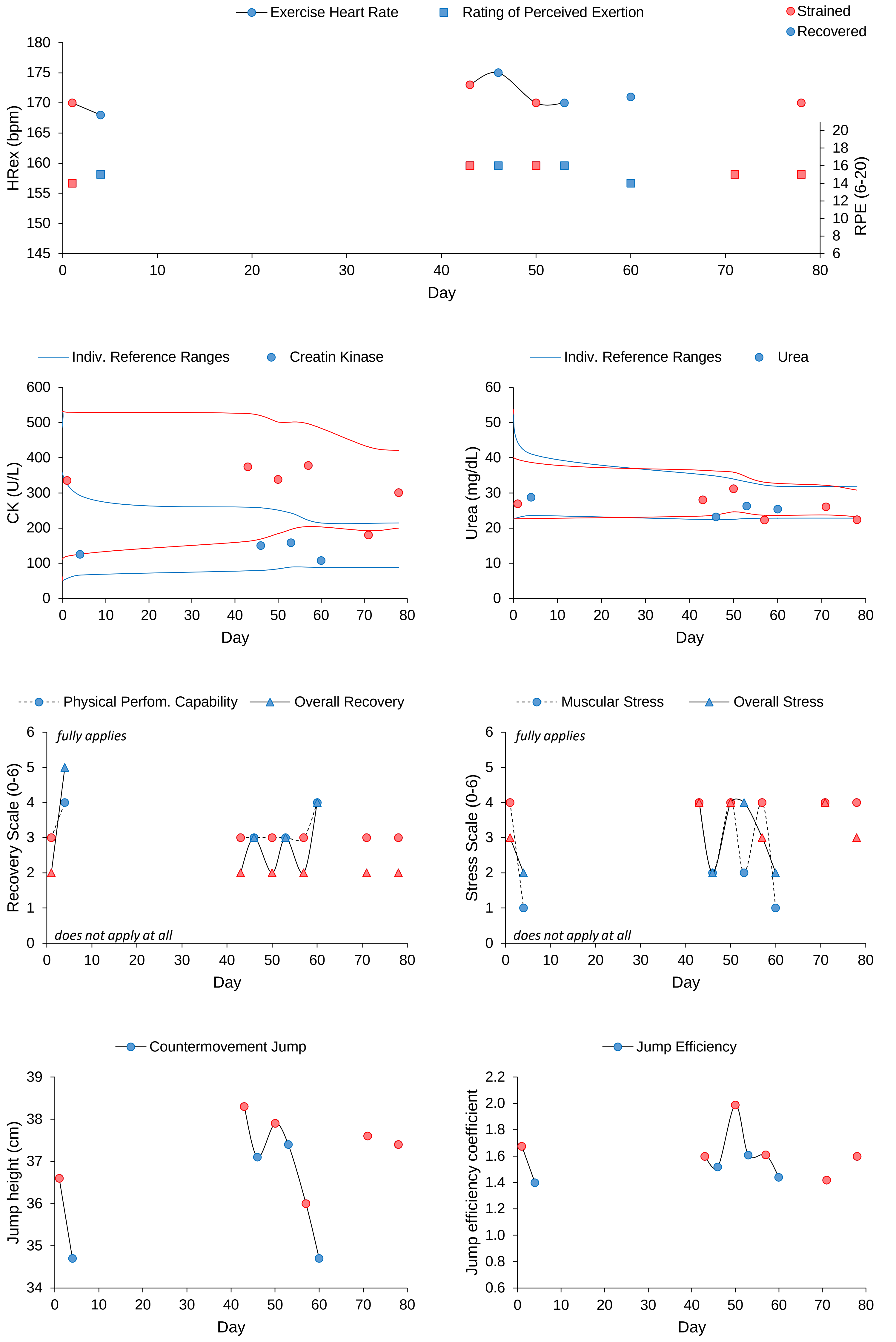

Individual data during the observational period. Blue dots: 'recovered' state (Mondays), red dots: 'strained' state (Fridays). Lines between data points are interrupted if measurements are not available or missing. HRex: exercise heart rate, RPE: rating of perceived exertion, CK: creatine kinase. Plots for CK and Urea also display individualized reference ranges for 'strained' (red lines) and 'recovered' data (blue lines) (95% confidence intervals), see Hecksteden et al. (2017) for reference and spreadsheets: https://doi.org/10.1123/ijspp.2016-0120

Player C

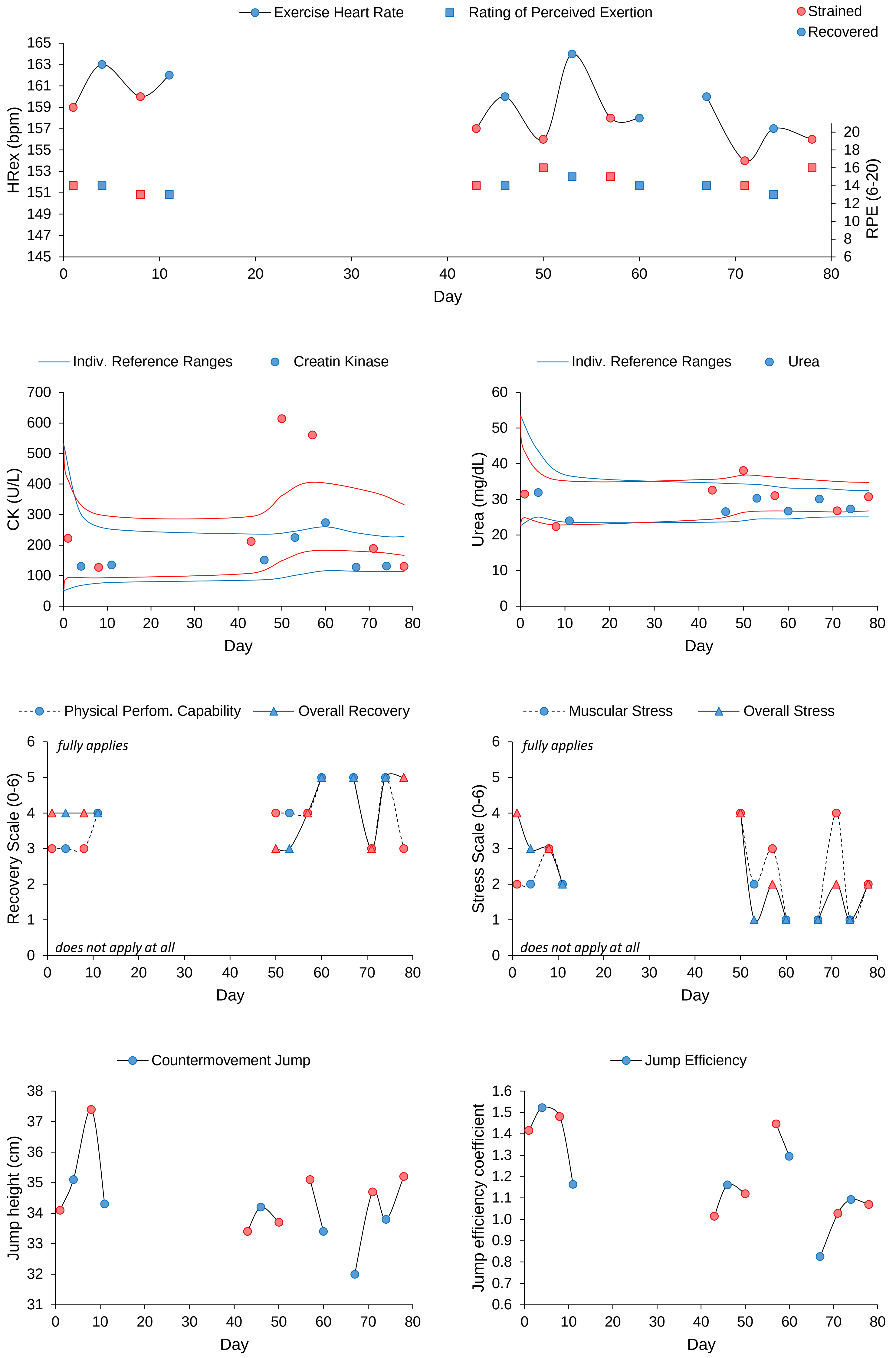

Individual data during the observational period. Blue dots: 'recovered' state (Mondays), red dots: 'strained' state (Fridays). Lines between data points are interrupted if measurements are not available or missing. HRex: exercise heart rate, RPE: rating of perceived exertion, CK: creatine kinase. Plots for CK and Urea also display individualized reference ranges for 'strained' (red lines) and 'recovered' data (blue lines) (95% confidence intervals), see Hecksteden et al. (2017) for reference and spreadsheets: https://doi.org/10.1123/ijspp.2016-0120

Player D

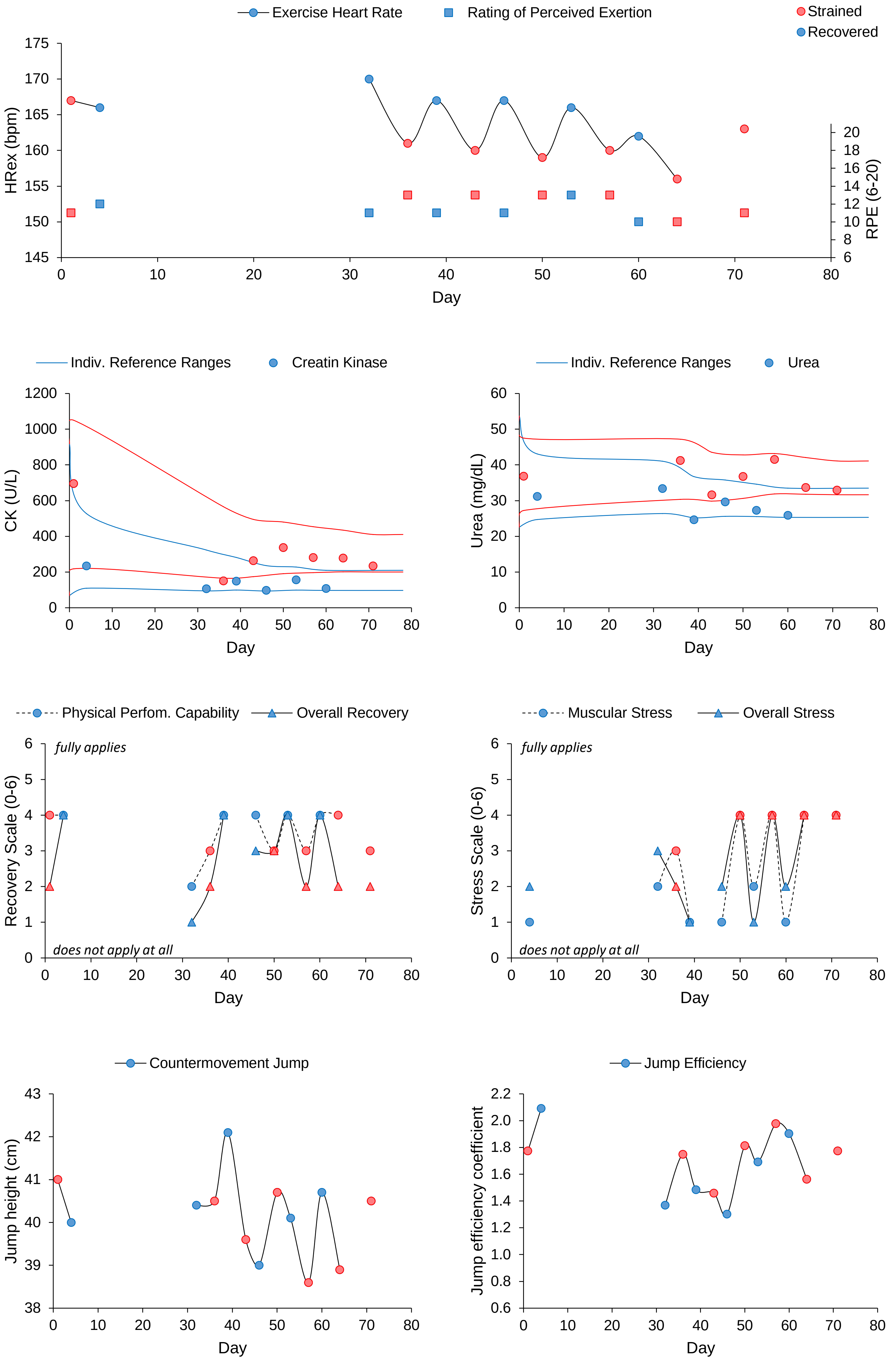

Individual data during the observational period. Blue dots: 'recovered' state (Mondays), red dots: 'strained' state (Fridays). Lines between data points are interrupted if measurements are not available or missing. HRex: exercise heart rate, RPE: rating of perceived exertion, CK: creatine kinase. Plots for CK and Urea also display individualized reference ranges for 'strained' (red lines) and 'recovered' data (blue lines) (95% confidence intervals), see Hecksteden et al. (2017) for reference and spreadsheets: https://doi.org/10.1123/ijspp.2016-0120

Player E

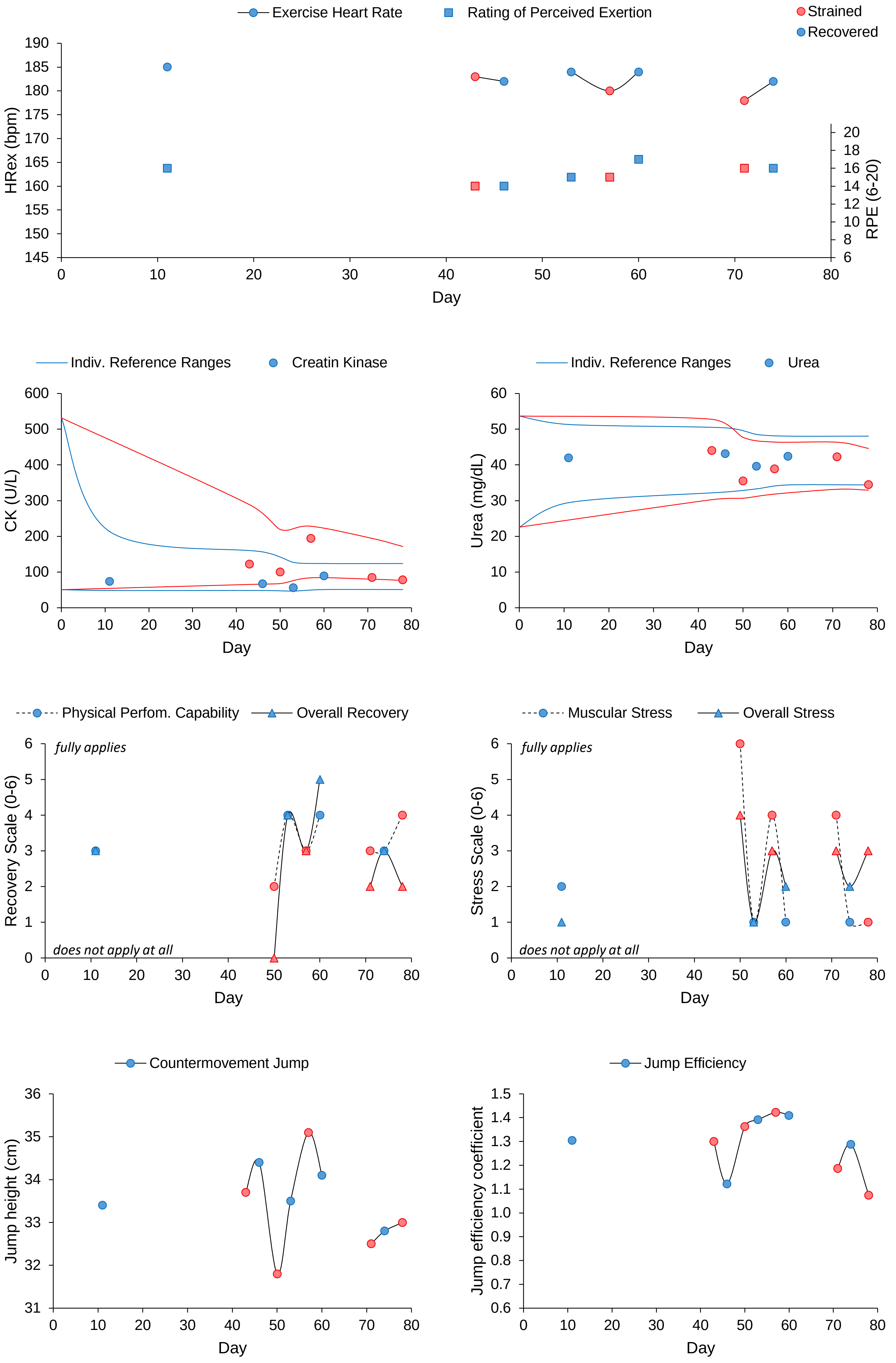

Individual data during the observational period. Blue dots: 'recovered' state (Mondays), red dots: 'strained' state (Fridays). Lines between data points are interrupted if measurements are not available or missing. HRex: exercise heart rate, RPE: rating of perceived exertion, CK: creatine kinase. Plots for CK and Urea also display individualized reference ranges for 'strained' (red lines) and 'recovered' data (blue lines) (95% confidence intervals), see Hecksteden et al. (2017) for reference and spreadsheets: https://doi.org/10.1123/ijspp.2016-0120

Player F

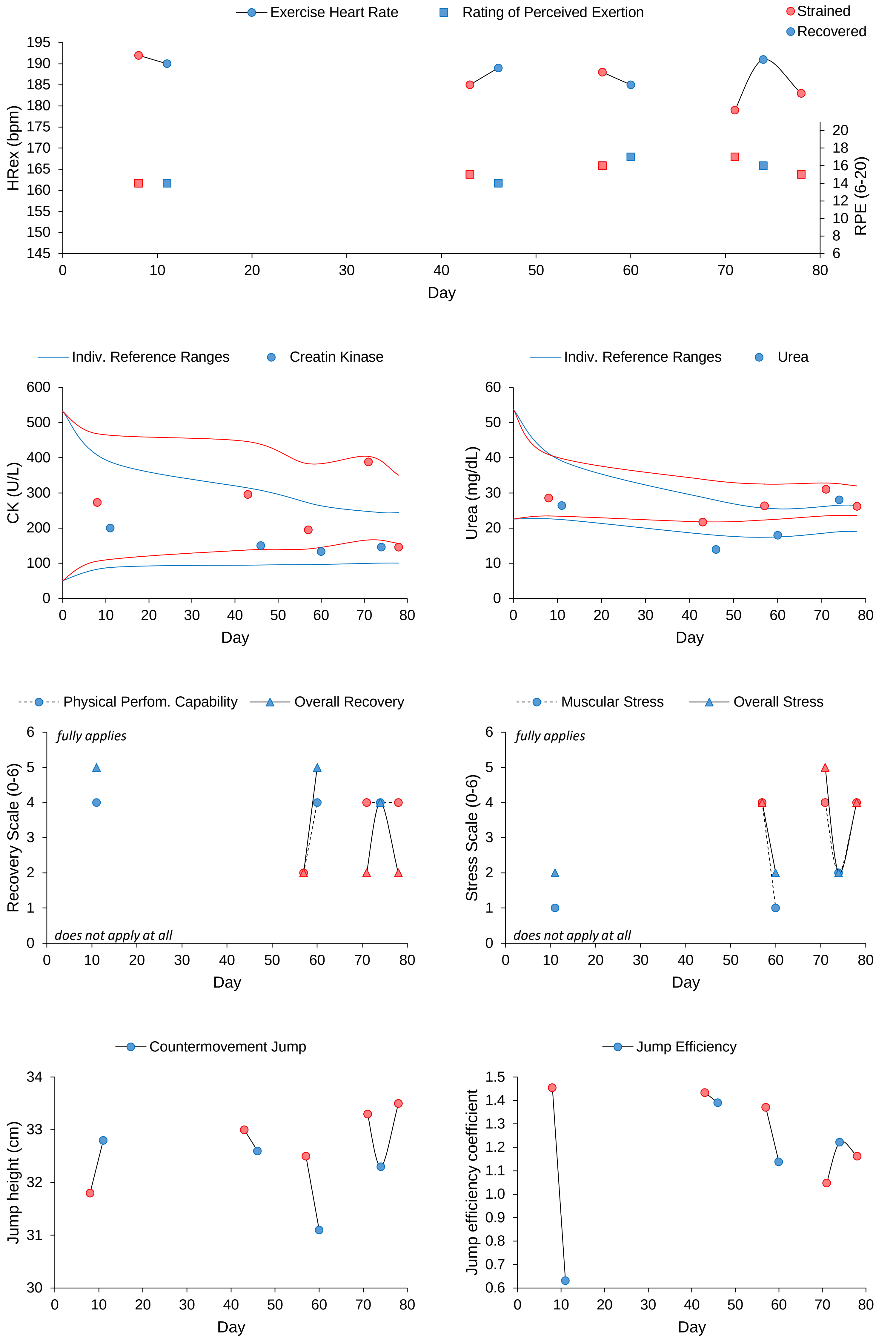

Individual data during the observational period. Blue dots: 'recovered' state (Mondays), red dots: 'strained' state (Fridays). Lines between data points are interrupted if measurements are not available or missing. HRex: exercise heart rate, RPE: rating of perceived exertion, CK: creatine kinase. Plots for CK and Urea also display individualized reference ranges for 'strained' (red lines) and 'recovered' data (blue lines) (95% confidence intervals), see Hecksteden et al. (2017) for reference and spreadsheets: https://doi.org/10.1123/ijspp.2016-0120

Player G

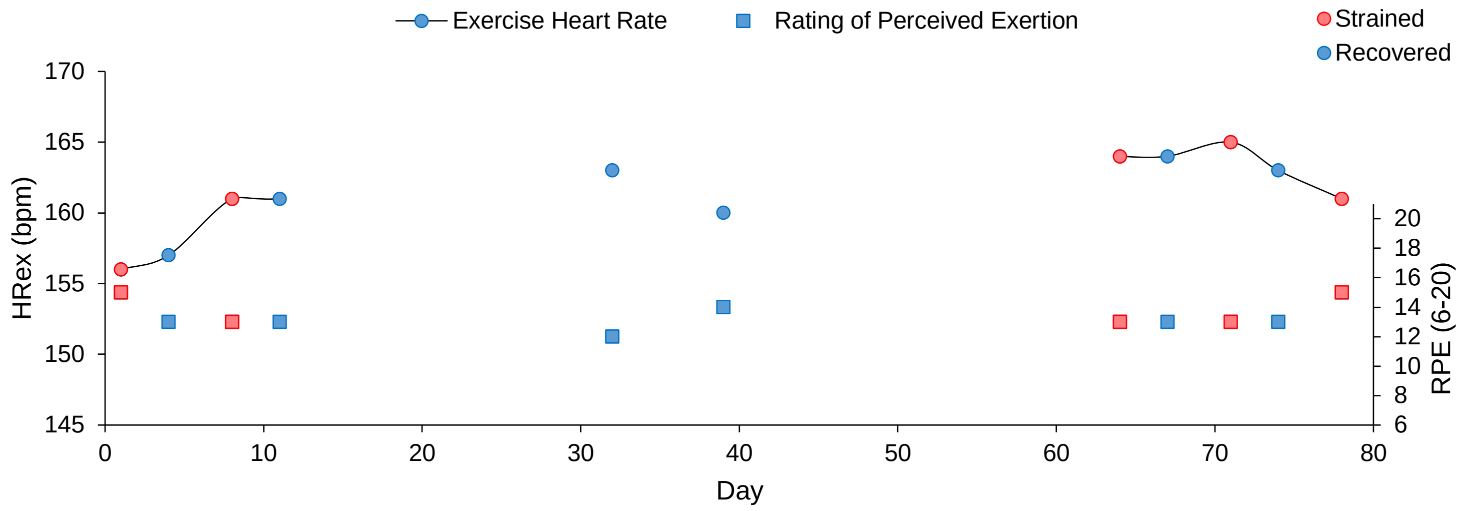

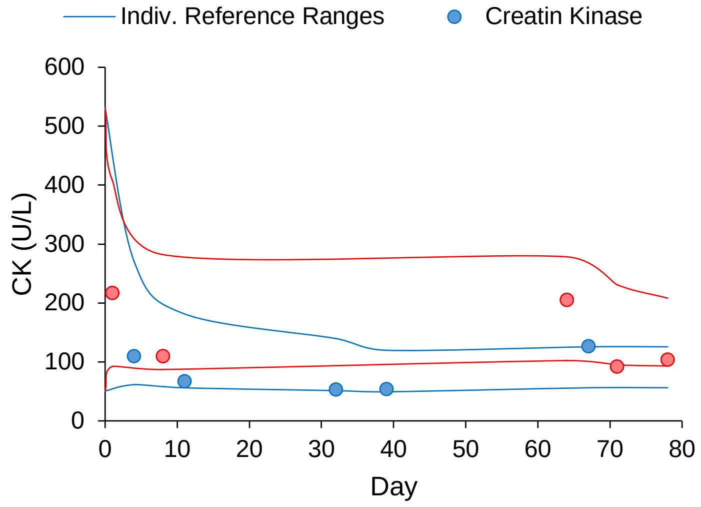

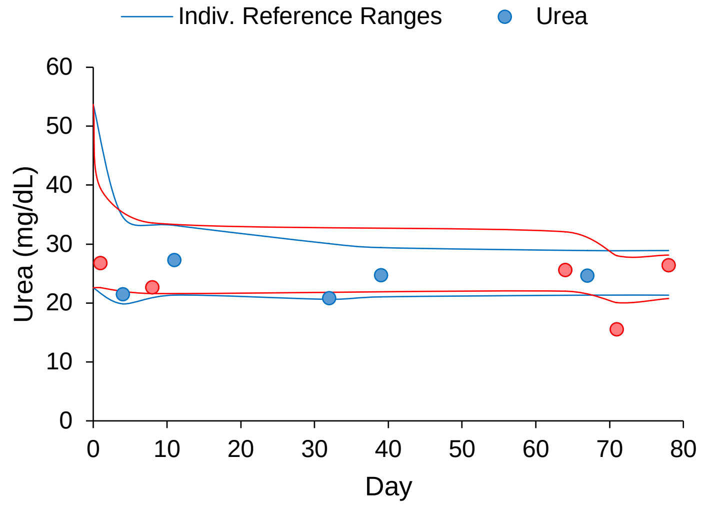

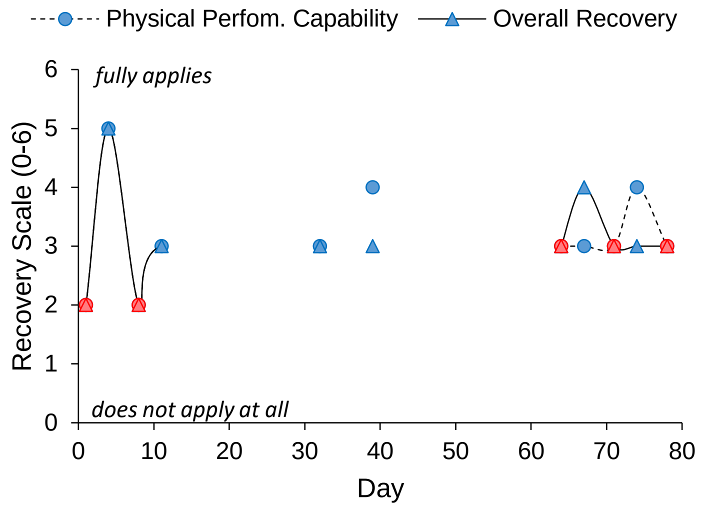

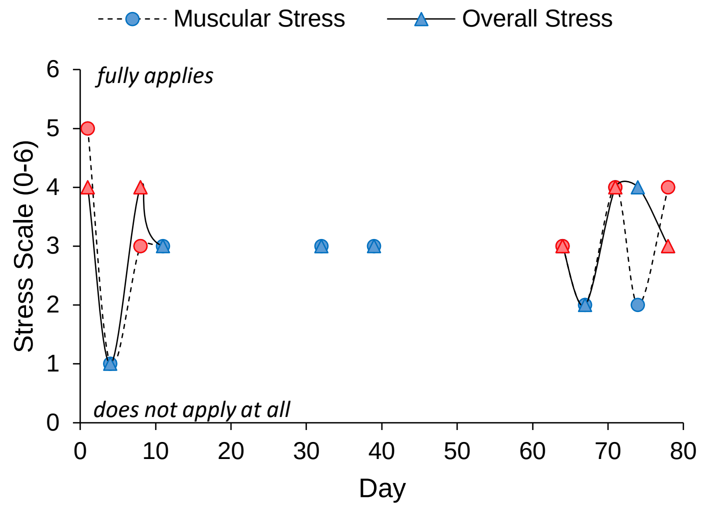

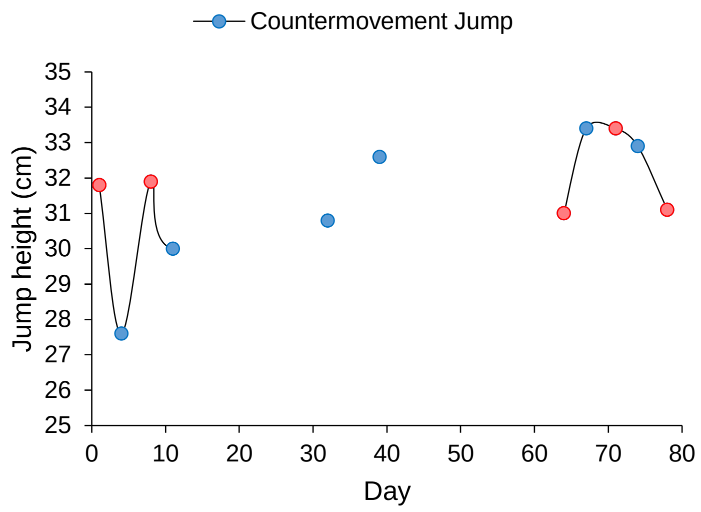

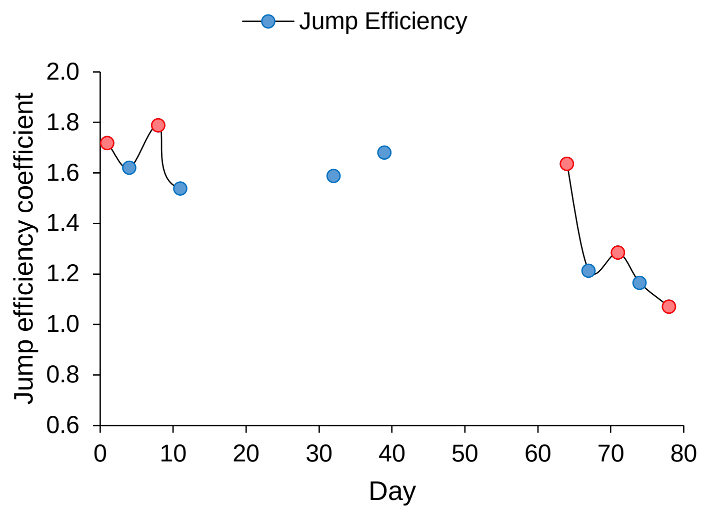

Individual data during the observational period. Blue dots: 'recovered' state (Mondays), red dots: 'strained' state (Fridays). Lines between data points are interrupted if measurements are not available or missing. HRex: exercise heart rate, RPE: rating of perceived exertion, CK: creatine kinase. Plots for CK and Urea also display individualized reference ranges for 'strained' (red lines) and 'recovered' data (blue lines) (95% confidence intervals), see Hecksteden et al. (2017) for reference and spreadsheets: https://doi.org/10.1123/ijspp.2016-0120

Player H

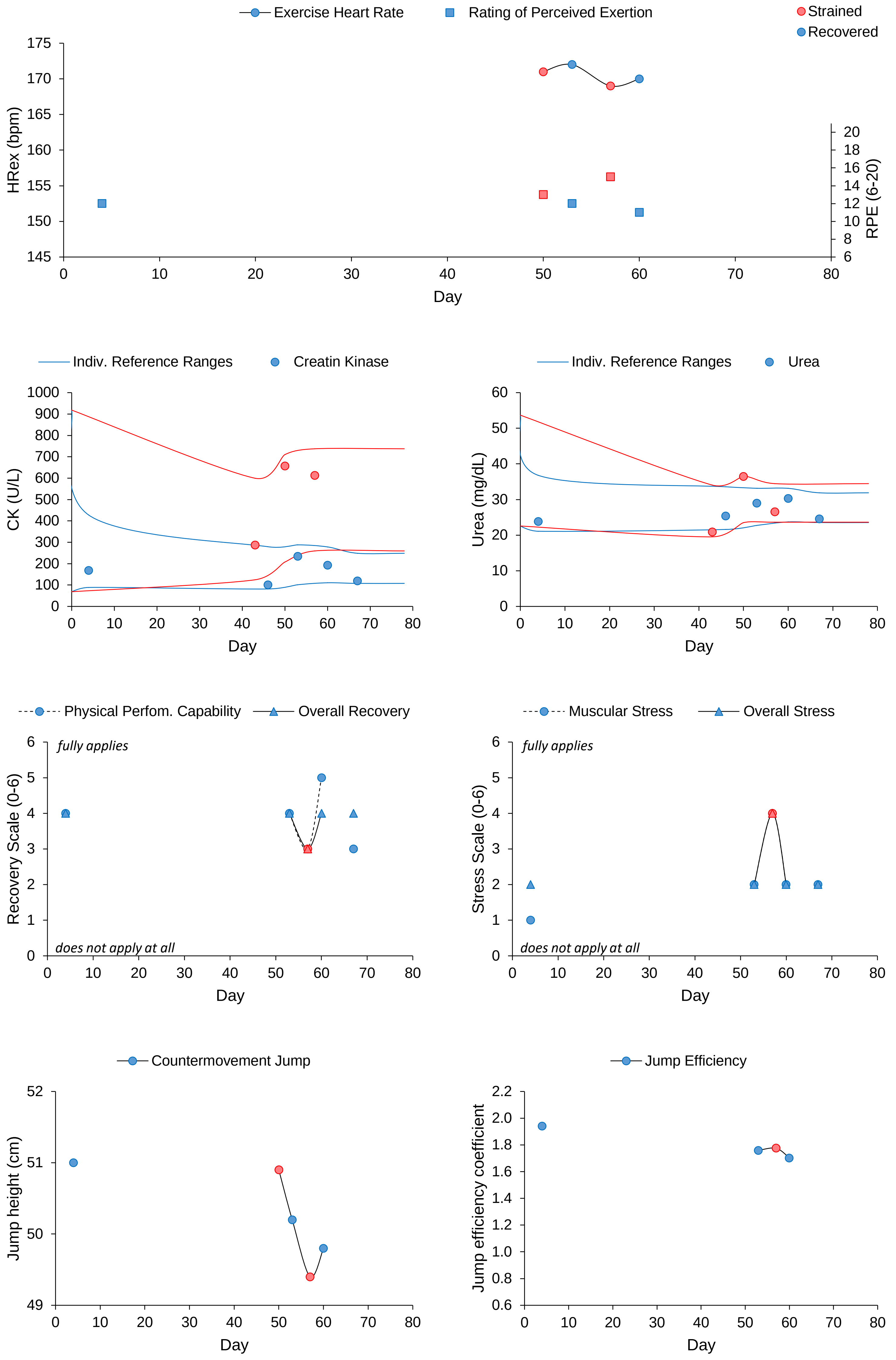

Individual data during the observational period. Blue dots: 'recovered' state (Mondays), red dots: 'strained' state (Fridays). Lines between data points are interrupted if measurements are not available or missing. HRex: exercise heart rate, RPE: rating of perceived exertion, CK: creatine kinase. Plots for CK and Urea also display individualized reference ranges for 'strained' (red lines) and 'recovered' data (blue lines) (95% confidence intervals), see Hecksteden et al. (2017) for reference and spreadsheets: https://doi.org/10.1123/ijspp.2016-0120

Player I

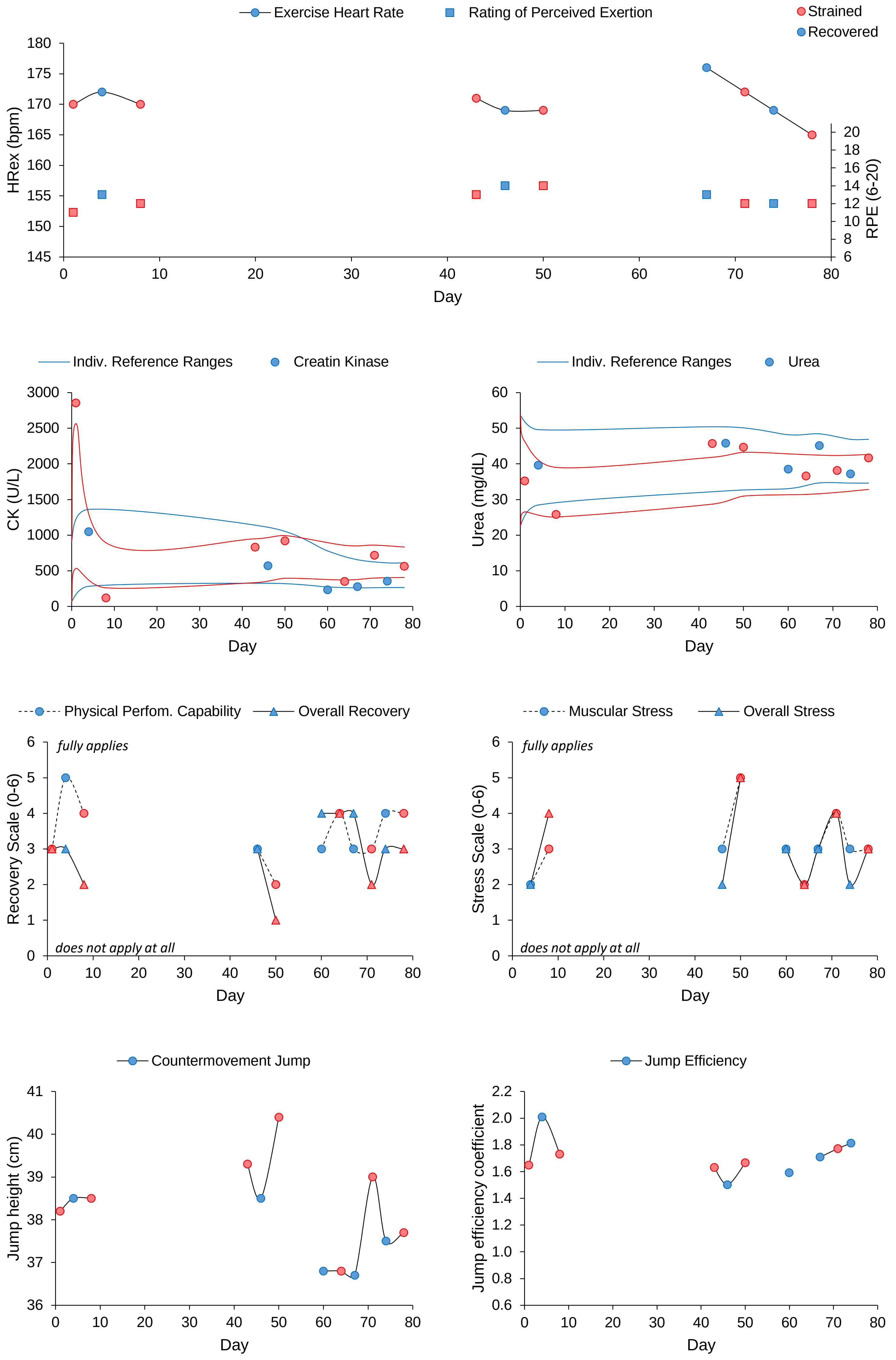

Individual data during the observational period. Blue dots: 'recovered' state (Mondays), red dots: 'strained' state (Fridays). Lines between data points are interrupted if measurements are not available or missing. HRex: exercise heart rate, RPE: rating of perceived exertion, CK: creatine kinase. Plots for CK and Urea also display individualized reference ranges for 'strained' (red lines) and 'recovered' data (blue lines) (95% confidence intervals), see Hecksteden et al. (2017) for reference and spreadsheets: https://doi.org/10.1123/ijspp.2016-0120

Player J

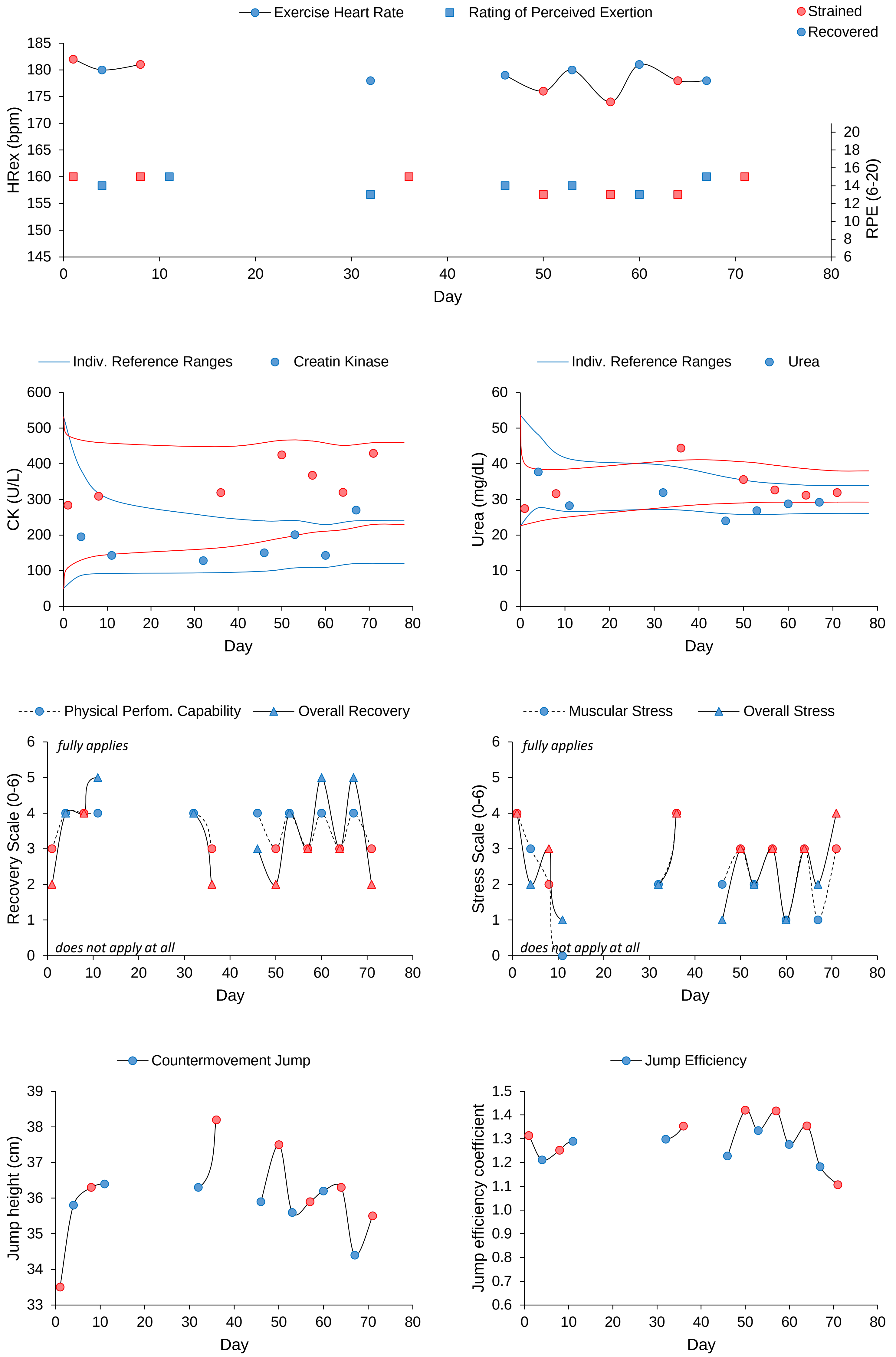

Individual data during the observational period. Blue dots: 'recovered' state (Mondays), red dots: 'strained' state (Fridays). Lines between data points are interrupted if measurements are not available or missing. HRex: exercise heart rate, RPE: rating of perceived exertion, CK: creatine kinase. Plots for CK and Urea also display individualized reference ranges for 'strained' (red lines) and 'recovered' data (blue lines) (95% confidence intervals), see Hecksteden et al. (2017) for reference and spreadsheets: https://doi.org/10.1123/ijspp.2016-0120
