# Supplementary figures and images for: Monitoring training and recovery responses with heart rate measures during standardized warm-up in elite badminton players
Source: PLoS One. 2020 Dec 21;15(12):e0244412. doi: 10.1371/journal.pone.0244412 (PMC7751974; doi:10.1371/journal.pone.0244412)

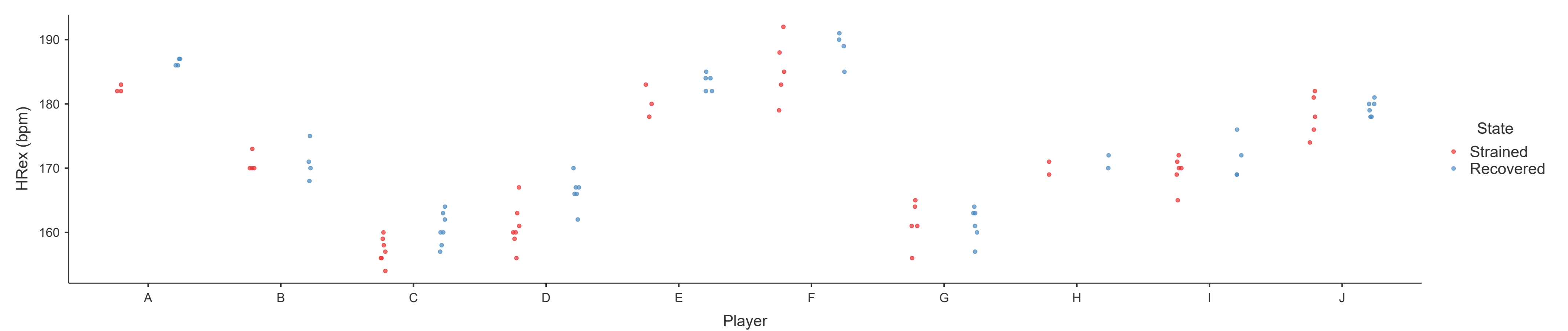

Supplement: S1 Fig — Plot displays individual HRex for recovered (blue) and strained (red) state during the 12-week study period. (PDF) [file pone.0244412.s003.pdf]

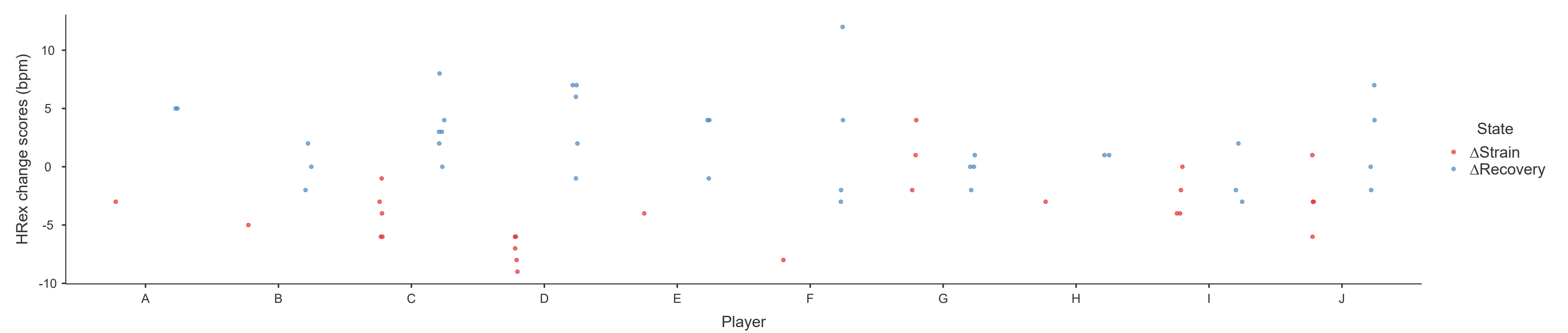

Supplement: S2 Fig — Plot displays individual change scores in HRex following recovery (ΔRecovery, blue) and following training strain (ΔStrain, red) during the 12-week study period. (PDF) [file pone.0244412.s004.pdf]
