# Supplementary material for: Monitoring training and recovery responses with heart rate measures during standardized warm-up in elite badminton players
Source: PLoS One. 2020 Dec 21;15(12):e0244412. doi: 10.1371/journal.pone.0244412 (PMC7751974; doi:10.1371/journal.pone.0244412)
Supplement: S1 Table — Individual data are summarized for recovered and strained state. Group data summarizes mean individual data. (PDF) [file pone.0244412.s005.pdf]

**S1 Table. Summary of individual monitoring data.**
Individual data are summarized for recovered and strained state. Group data summarizes mean individual data.

| Player | A | | B | | C | | D | | E | | F | | G | | H | | I | | J | | Overall | |
|---|---|---|---|---|---|---|---|---|---|---|---|---|---|---|---|---|---|---|---|---|---|---|
| State | Rec | Strain | Rec | Strain | Rec | Strain | Rec | Strain | Rec | Strain | Rec | Strain | Rec | Strain | Rec | Strain | Rec | Strain | Rec | Strain | Rec | Strain |
| **Natural Logarithm of Creatine Kinase (U/L)** | | | | | | | | | | | | | | | | | | | | | | |
| **Tests** | 5 | 5 | 4 | 6 | 7 | 7 | 6 | 7 | 4 | 5 | 4 | 5 | 5 | 5 | 5 | 3 | 5 | 7 | 7 | 7 | 52 | 57 |
| **Mean** | 4.78 | 5.30 | 4.90 | 5.73 | 5.08 | 5.50 | 4.91 | 5.67 | 4.26 | 4.70 | 5.05 | 5.50 | 4.34 | 4.91 | 5.05 | 6.19 | 6.05 | 6.44 | 5.14 | 5.85 | 4.95 | 5.58 |
| **SD** | 0.11 | 0.40 | 0.17 | 0.28 | 0.31 | 0.64 | 0.33 | 0.46 | 0.19 | 0.36 | 0.18 | 0.38 | 0.41 | 0.41 | 0.35 | 0.46 | 0.61 | 0.97 | 0.26 | 0.16 | 0.49 | 0.53 |
| **Diff.** | 0.52 | | 0.84 | | 0.42 | | 0.76 | | 0.44 | | 0.46 | | 0.57 | | 1.14 | | 0.39 | | 0.71 | | 0.62 ± 0.24 | |
| ***d*** | 1.60 | | 2.56 | | 1.27 | | 2.34 | | 1.34 | | 1.40 | | 1.75 | | 3.49 | | 1.18 | | 2.17 | | 1.91 ± 0.73 | |
| **Creatine Kinase (U/L)** | | | | | | | | | | | | | | | | | | | | | | |
| **Antilog** | 119 | 201 | 134 | 309 | 161 | 244 | 135 | 290 | 71 | 109 | 155 | 245 | 77 | 136 | 156 | 487 | 425 | 625 | 170 | 347 | 142 | 265 |
| **$SD_{antilog}$** | [107, 133] | [134, 300] | [112, 159] | [234, 408] | [118, 218] | [129, 462] | [97, 188] | [183, 460] | [58, 86] | [76, 157] | [130, 185] | [168, 359] | [51, 115] | [91, 204] | [110, 220] | [308, 772] | [232, 779] | [237, 1646] | [131, 222] | [295, 407] | [87, 232] | [156, 450] |
| **$Diff_{antilog}$** | 82 | | 175 | | 83 | | 155 | | 39 | | 90 | | 59 | | 332 | | 200 | | 176 | | 123 | |
| **Natural Logarithm of Urea (mg/dL)** | | | | | | | | | | | | | | | | | | | | | | |
| **Tests** | 5 | 5 | 4 | 6 | 7 | 7 | 6 | 7 | 4 | 5 | 4 | 5 | 5 | 5 | 5 | 3 | 5 | 7 | 7 | 7 | 52 | 57 |
| **Mean** | 2.92 | 3.11 | 3.25 | 3.26 | 3.33 | 3.40 | 3.35 | 3.59 | 3.73 | 3.66 | 3.03 | 3.28 | 3.17 | 3.13 | 3.28 | 3.31 | 3.72 | 3.63 | 3.38 | 3.50 | 3.32 | 3.39 |
| **SD** | 0.13 | 0.26 | 0.09 | 0.13 | 0.10 | 0.17 | 0.12 | 0.11 | 0.04 | 0.11 | 0.33 | 0.13 | 0.11 | 0.23 | 0.11 | 0.28 | 0.09 | 0.19 | 0.14 | 0.15 | 0.26 | 0.20 |
| **Diff.** | 0.19 | | 0.01 | | 0.07 | | 0.24 | | -0.07 | | 0.25 | | -0.03 | | 0.03 | | -0.09 | | 0.13 | | 0.07 ± 0.12 | |
| ***d*** | 1.38 | | 0.04 | | 0.52 | | 1.73 | | -0.52 | | 1.79 | | -0.22 | | 0.20 | | -0.64 | | 0.92 | | 0.52 ± 0.90 | |
| **Urea (mg/dL)** | | | | | | | | | | | | | | | | | | | | | | |
| **Antilog** | 19 | 22 | 26 | 26 | 28 | 30 | 29 | 36 | 42 | 39 | 21 | 27 | 24 | 23 | 26 | 27 | 41 | 38 | 29 | 33 | 28 | 30 |
| **$SD_{antilog}$** | [16, 21] | [17, 29] | [24, 28] | [23, 30] | [25, 31] | [25, 36] | [25, 32] | [33, 40] | [40, 43] | [35, 43] | [15, 29] | [23, 30] | [21, 26] | [18, 29] | [24, 29] | [21, 36] | [37, 45] | [31, 46] | [25, 34] | [29, 39] | [21, 36] | [24, 36] |
| **$Diff_{antilog}$** | 4 | | 0 | | 2 | | 8 | | -3 | | 6 | | -1 | | 1 | | -3 | | 4 | | 2 | |

**S1 Table.** Continued

| Player | A | | B | | C | | D | | E | | F | | G | | H | | I | | J | | Overall | |
|---|---|---|---|---|---|---|---|---|---|---|---|---|---|---|---|---|---|---|---|---|---|---|
| **State** | Rec | Strain | Rec | Strain | Rec | Strain | Rec | Strain | Rec | Strain | Rec | Strain | Rec | Strain | Rec | Strain | Rec | Strain | Rec | Strain | Rec | Strain |
| **Short Recovery and Stress Scale - Physical Performance Capability (0-6)** | | | | | | | | | | | | | | | | | | | | | | |
| **Tests** | 4 | 3 | 4 | 6 | 6 | 6 | 6 | 6 | 4 | 4 | 3 | 3 | 6 | 5 | 4 | 1 | 5 | 6 | 7 | 7 | 49 | 47 |
| **Mean** | 3.5 | 3.3 | 3.5 | 3.0 | 4.3 | 3.3 | 3.7 | 3.3 | 3.5 | 3.0 | 4.0 | 3.3 | 3.7 | 2.6 | 4.0 | 3.0 | 3.6 | 3.3 | 4.0 | 3.1 | 3.8 | 3.1 |
| **SD** | 0.6 | 0.6 | 0.6 | 0.0 | 0.8 | 0.5 | 0.8 | 0.5 | 0.6 | 0.8 | 0.0 | 1.2 | 0.8 | 0.5 | 0.8 | | 0.9 | 0.8 | 0.0 | 0.4 | 0.3 | 0.2 |
| **Diff.** | -0.2 | | -0.5 | | -1.0 | | -0.3 | | -0.5 | | -0.7 | | -1.1 | | -1.0 | | -0.3 | | -0.9 | | -0.6 ± 0.3 | |
| ***d*** | -0.24 | | -0.73 | | -1.46 | | -0.49 | | -0.73 | | -0.98 | | -1.56 | | -1.46 | | -0.39 | | -1.25 | | -0.93 ± 0.48 | |
| **Short Recovery and Stress Scale - Overall Recovery (0-6)** | | | | | | | | | | | | | | | | | | | | | | |
| **Tests** | 4 | 3 | 4 | 6 | 6 | 6 | 6 | 6 | 4 | 4 | 3 | 3 | 6 | 5 | 4 | 1 | 5 | 6 | 7 | 7 | 49 | 47 |
| **Mean** | 3.3 | 2.3 | 3.8 | 2.0 | 4.3 | 3.8 | 3.3 | 2.2 | 3.8 | 1.8 | 4.7 | 2.0 | 3.5 | 2.6 | 4.0 | 3.0 | 3.4 | 2.5 | 4.3 | 2.6 | 3.8 | 2.5 |
| **SD** | 1.0 | 0.6 | 1.0 | 0.0 | 0.8 | 0.8 | 1.2 | 0.4 | 1.0 | 1.3 | 0.6 | 0.0 | 0.8 | 0.5 | 0.0 | | 0.5 | 1.0 | 0.8 | 0.8 | 0.5 | 0.6 |
| **Diff.** | -0.9 | | -1.8 | | -0.5 | | -1.2 | | -2.0 | | -2.7 | | -0.9 | | -1.0 | | -0.9 | | -1.7 | | -1.4 ± 0.7 | |
| ***d*** | -1.09 | | -2.08 | | -0.59 | | -1.38 | | -2.37 | | -3.16 | | -1.07 | | -1.19 | | -1.07 | | -2.03 | | -1.60 ± 0.78 | |
| **Short Recovery and Stress Scale - Muscular Stress (0-6)** | | | | | | | | | | | | | | | | | | | | | | |
| **Tests** | 4 | 3 | 4 | 6 | 6 | 6 | 6 | 5 | 4 | 4 | 3 | 3 | 6 | 5 | 4 | 1 | 5 | 5 | 7 | 7 | 49 | 45 |
| **Mean** | 2.3 | 3.7 | 1.5 | 4.0 | 1.5 | 3.0 | 1.3 | 3.8 | 1.3 | 3.8 | 1.3 | 4.0 | 2.3 | 3.8 | 1.8 | 4.0 | 2.8 | 3.4 | 1.6 | 3.1 | 1.8 | 3.7 |
| **SD** | 0.5 | 0.6 | 0.6 | 0.0 | 0.5 | 0.9 | 0.5 | 0.4 | 0.5 | 2.1 | 0.6 | 0.0 | 0.8 | 0.8 | 0.5 | | 0.4 | 1.1 | 1.0 | 0.7 | 0.5 | 0.4 |
| **Diff.** | 1.4 | | 2.5 | | 1.5 | | 2.5 | | 2.5 | | 2.7 | | 1.5 | | 2.3 | | 0.6 | | 1.6 | | 1.9 ± 0.7 | |
| ***d*** | 2.17 | | 3.83 | | 2.30 | | 3.78 | | 3.83 | | 4.09 | | 2.25 | | 3.45 | | 0.92 | | 2.41 | | 2.90 ± 1.04 | |
| **Short Recovery and Stress Scale - Overall Stress (0-6)** | | | | | | | | | | | | | | | | | | | | | | |
| **Tests** | 4 | 3 | 4 | 6 | 6 | 6 | 6 | 5 | 4 | 4 | 3 | 3 | 6 | 5 | 4 | 1 | 5 | 5 | 7 | 7 | 49 | 45 |
| **Mean** | 3.0 | 3.7 | 2.5 | 3.5 | 1.5 | 2.8 | 1.8 | 3.6 | 1.5 | 3.3 | 2.0 | 4.3 | 2.7 | 3.6 | 2.0 | 4.0 | 2.4 | 3.6 | 1.6 | 3.4 | 2.1 | 3.6 |
| **SD** | 1.2 | 1.2 | 1.0 | 0.5 | 0.8 | 1.0 | 0.8 | 0.9 | 0.6 | 0.5 | 0.0 | 0.6 | 1.0 | 0.5 | 0.0 | | 0.5 | 1.1 | 0.5 | 0.5 | 0.5 | 0.4 |
| **Diff.** | 0.7 | | 1.0 | | 1.3 | | 1.8 | | 1.8 | | 2.3 | | 0.9 | | 2.0 | | 1.2 | | 1.9 | | 1.5 ± 0.5 | |
| ***d*** | 0.88 | | 1.31 | | 1.75 | | 2.32 | | 2.30 | | 3.07 | | 1.23 | | 2.63 | | 1.58 | | 2.44 | | 1.95 ± 0.70 | |

**S1 Table.** Continued

| Player | A | | B | | C | | D | | E | | F | | G | | H | | I | | J | | Overall | |
|---|---|---|---|---|---|---|---|---|---|---|---|---|---|---|---|---|---|---|---|---|---|---|
| **State** | Rec | Strain | Rec | Strain | Rec | Strain | Rec | Strain | Rec | Strain | Rec | Strain | Rec | Strain | Rec | Strain | Rec | Strain | Rec | Strain | Rec | Strain |
| **Countermovement Jump Height (cm)** | | | | | | | | | | | | | | | | | | | | | | |
| **Tests** | 5 | 5 | 4 | 6 | 6 | 7 | 6 | 7 | 5 | 5 | 4 | 5 | 6 | 5 | 3 | 2 | 5 | 7 | 7 | 7 | 51 | 56 |
| **Mean** | 29.2 | 29.2 | 36.0 | 37.3 | 33.8 | 34.8 | 40.4 | 40.0 | 33.6 | 33.2 | 32.2 | 32.8 | 31.2 | 31.8 | 50.3 | 50.2 | 37.6 | 38.6 | 35.8 | 36.2 | 36.0 | 36.4 |
| **SD** | 0.9 | 1.0 | 1.5 | 0.9 | 1.0 | 1.3 | 1.0 | 0.9 | 0.6 | 1.3 | 0.8 | 0.7 | 2.2 | 1.0 | 0.6 | 1.1 | 0.9 | 1.2 | 0.7 | 1.5 | 6.0 | 5.8 |
| **Diff.** | 0.0 | | 1.3 | | 1.0 | | -0.4 | | -0.4 | | 0.6 | | 0.6 | | -0.2 | | 1.0 | | 0.4 | | 0.4 ± 0.6 | |
| ***d*** | 0.00 | | 1.14 | | 0.87 | | -0.36 | | -0.36 | | 0.54 | | 0.54 | | -0.16 | | 0.83 | | 0.32 | | 0.34 ± 0.54 | |
| **Jump Efficiency Coefficient (index)** | | | | | | | | | | | | | | | | | | | | | | |
| **Tests** | 5 | 5 | 4 | 6 | 6 | 7 | 6 | 7 | 5 | 5 | 4 | 5 | 6 | 5 | 3 | 1 | 5 | 5 | 7 | 7 | 51 | 53 |
| **Mean** | 1.13 | 1.14 | 1.49 | 1.65 | 1.18 | 1.23 | 1.64 | 1.73 | 1.30 | 1.27 | 1.10 | 1.29 | 1.47 | 1.50 | 1.80 | 1.78 | 1.73 | 1.69 | 1.26 | 1.32 | 1.41 | 1.46 |
| **SD** | 0.17 | 0.17 | 0.09 | 0.19 | 0.23 | 0.21 | 0.31 | 0.17 | 0.11 | 0.14 | 0.33 | 0.18 | 0.22 | 0.31 | 0.13 | | 0.20 | 0.06 | 0.05 | 0.11 | 0.25 | 0.24 |
| **Diff.** | 0.02 | | 0.16 | | 0.05 | | 0.09 | | -0.03 | | 0.20 | | 0.03 | | -0.03 | | -0.04 | | 0.06 | | 0.05 ± 0.08 | |
| ***d*** | 0.08 | | 0.77 | | 0.23 | | 0.43 | | -0.17 | | 0.97 | | 0.15 | | -0.13 | | -0.17 | | 0.27 | | 0.24 ± 0.39 | |
| **Exercise Heart Rate (bpm)** | | | | | | | | | | | | | | | | | | | | | | |
| **Tests** | 4 | 3 | 4 | 4 | 7 | 7 | 6 | 7 | 5 | 3 | 4 | 5 | 6 | 5 | 2 | 2 | 4 | 6 | 6 | 5 | 48 | 47 |
| **Mean** | 186.5 | 182.3 | 171.0 | 170.8 | 160.6 | 157.1 | 166.3 | 160.9 | 183.4 | 180.3 | 188.8 | 185.4 | 161.3 | 161.4 | 171.0 | 170.0 | 171.5 | 169.5 | 179.3 | 178.2 | 174.0 | 171.6 |
| **SD** | 0.6 | 0.6 | 2.9 | 1.5 | 2.6 | 2.0 | 2.6 | 3.4 | 1.3 | 2.5 | 2.6 | 4.9 | 2.6 | 3.5 | 1.4 | 1.4 | 3.3 | 2.4 | 1.2 | 3.3 | 10.1 | 9.8 |
| **Diff.** | -4.2 | | -0.2 | | -3.5 | | -5.4 | | -3.1 | | -3.4 | | 0.1 | | -1.0 | | -2.0 | | -1.1 | | -2.4 ± 1.8 | |
| ***d*** | -1.81 | | -0.09 | | -1.51 | | -2.33 | | -1.34 | | -1.47 | | 0.04 | | -0.43 | | -0.86 | | -0.48 | | -1.03 ± 0.79 | |
| **Rating of Perceived Exertion (6-20)** | | | | | | | | | | | | | | | | | | | | | | |
| **Tests** | 5 | 4 | 4 | 5 | 7 | 7 | 6 | 7 | 5 | 3 | 4 | 5 | 6 | 5 | 3 | 2 | 4 | 6 | 7 | 7 | 51 | 51 |
| **Mean** | 15.8 | 15.8 | 15.3 | 15.2 | 13.9 | 14.6 | 11.3 | 12.0 | 15.6 | 15.0 | 15.3 | 15.4 | 13.0 | 13.8 | 11.7 | 14.0 | 13.0 | 12.3 | 14.0 | 14.1 | 13.9 | 14.2 |
| **SD** | 1.1 | 1.0 | 1.0 | 0.8 | 0.7 | 1.1 | 1.0 | 1.3 | 1.1 | 1.0 | 1.5 | 1.1 | 0.6 | 1.1 | 0.6 | 1.4 | 0.8 | 1.0 | 0.8 | 1.1 | 1.6 | 1.3 |
| **Diff.** | -0.1 | | -0.1 | | 0.7 | | 0.7 | | -0.6 | | 0.2 | | 0.8 | | 2.3 | | -0.7 | | 0.1 | | 0.3 ± 0.9 | |
| ***d*** | -0.05 | | -0.05 | | 0.75 | | 0.71 | | -0.64 | | 0.16 | | 0.85 | | 2.47 | | -0.71 | | 0.15 | | 0.36 ± 0.92 | |

Rec: 'recovered' state; Strain: 'strained' state; SD: standard deviation; Diff.: mean difference between recovered and strained state; *d*: standardized mean difference = mean difference divided by pooled within-player SD, Antilog: back-transformed means of lnCK and lnUrea, $\mathrm{Diff}_{antilog}$: difference between back-transformed means lnCK and lnUrea, $\mathrm{SD}_{antilog}$: back-transformed values for mean – SD and mean + SD for lnCK or lnUrea [mean – SD, mean + SD].
Overall: means, mean differences and standardized mean differences are presented as grand means and between-player SDs for all 10 players.
