# Supplementary material for: Monitoring training and recovery responses with heart rate measures during standardized warm-up in elite badminton players
Source: PLoS One. 2020 Dec 21;15(12):e0244412. doi: 10.1371/journal.pone.0244412 (PMC7751974; doi:10.1371/journal.pone.0244412)
Supplement: Attachment — Submitted filename: Rebuttal letter.docx [file pone.0244412.s007.docx]

Response to the Editor and the Reviewer

# Comments to the editor

**Editor:**

**Dear Dr. Schneider,**
**Thank you for submitting your manuscript to PLOS ONE. After careful consideration, we have decided that your manuscript does not meet our criteria for publication and must therefore be rejected. As you will see, some limits have been raised during the reviewing process.** **However, the major problem is that the entire text, results, figures and tables can be search open access in ResearchGate and SportRχiv, without peer-review and this migth be confonding for PLOS ONE.**

**I am sorry that we cannot be more positive on this occasion, but hope that you appreciate the reasons for this decision.**

**Yours sincerely,
Laurent Mourot
Academic Editor
PLOS ONE**

- Authors’ response:

Dear Dr. Mourot

Thank you for the quick editorial process and decision.

We believe that the decision to reject the manuscript for the reasons mentioned in the decision letter was a misunderstanding, as the main reason for rejection it was stated that “*the major problem is that the entire text, results, figures and tables can be search open access in ResearchGate and SportRχiv, without peer-review and this migth be confonding for PLOS ONE*”. This is in contrast to PLOS submission guidelines and policies. PLOS actively encourages authors to publish preprints prior to or upon submission. We believe that this issue has already been resolved in the process of our appeal request.

We also believe that the concerns raised by the reviewer have no substantial impact on the study results or conclusions. We provide detailed responses to the reviewer’s comments and have tried to make appropriate revision (in red).

Please find more detailed comments in the following.

a.) Preprint Publication:

We purposefully decided to publish the preprint and its underlying data in accordance to and because of the PLOS submission guidelines.

As stated in the submission guidelines, PLOS and its associated journals “*encourages authors to post preprints as a way to accelerate the dissemination of research*” (for reference: <https://journals.plos.org/plosone/s/preprints>). This includes the publication of the full manuscript in the pre-submission version, and all its accompanying results, material, and data. The open and findable dissemination of the manuscript and supporting material on repositories like SportRxiv or ResearchGate prior to submission and peer-review is an essential component of preprints. Based these PLOS guidelines we decided to publish a preprint to adhere to open science practices.

In addition, PLOS also “*require[s] authors to make all data necessary to replicate their study’s findings publicly available without restriction*” (<https://journals.plos.org/plosone/s/data-availability>). This was also done in accordance to PLOS data sharing policy and using the Open Science Framework as one of the PLOS recommended repositories (<https://journals.plos.org/plosone/s/recommended-repositories>).

b.) Reviewer comments:

We appreciate the critical comments by the reviewer. Dr. Solana-Tramunt raises some valid criticism. We believe that the concerned raised by the reviewer are in total of small magnitude and can be addressed with minor revision.

One main criticism of the reviewer was the non-availability of data (Question 2) and the inappropriate reporting guidelines and community standards by publishing (5. Comments to the Authors). Dr. Solana-Tramunt seems to have missed that the main dataset was uploaded as “Supporting Information” file and the full data and analysis documentation was deposited on the Open Science Framework (<https://doi.org/10.17605/OSF.IO/UP4HT>) and referenced within the submission process and within the manuscript. There might have been technical issues, so that she was not able to access the documents and information during the review process.

Content wise, the reviewer seemed to have missed an important analytical aspect:

The descriptive statistics for creatine kinase are indeed biased by pooling genders. As suggested by the reviewer, we therefore included descriptive statistics for creatine kinase (CK) und Urea for females only (Revisions in Table 1, page 11). However, the main analysis were within-subject comparisons with standardized mean differences, which reduces potential between subject effects in mean CK and Urea levels (e.g. due to gender, race). Thus, the described training strain-related effects in the surrogate measures creatine kinase and the subjective recovery and stress ratings were clearly and appropriately verified in every single subject. The criticism raised by Dr. Solana-Tramunt indeed concerns the descriptive statistics for creatine kinase, but it has little effect on the main results and no effect on the conclusions of our study for the analytical reasons mentioned above.

In this rebuttal letter we provided detailed responses to the reviewer’s comments and we believe that we have addressed the criticisms raised by the reviewer appropriately, while none of the criticism invalidates our methods or the main inferential results.

Kind regards,

Christoph Schneider (on behalf of the coauthors)

# Comments to the reviewer

Dear Dr. Solana-Tramunt,

Thank you for providing a critical and fast review of our manuscript. We believe that respective revisions (in red) help to improve the article. Please find our point-by-point response to your comments below.

We tried to respond appropriately to all concerns raised. Please also see the revisions made in the manuscript.

Kind regards,

Christoph Schneider

# Question 1

Is the manuscript technically sound, and do the data support the conclusions?

The manuscript must describe a technically sound piece of scientific research with data that supports the conclusions. Experiments must have been conducted rigorously, with appropriate controls, replication, and sample sizes. The conclusions must be drawn appropriately based on the data presented.

- **Reviewer #1: Partly**
- Authors’ response to question 1:

We will reply to all comments and criticism raised by the reviewer below.

# Question 2

Has the statistical analysis been performed appropriately and rigorously?

- **Reviewer #1: No**
- Authors’ response to question 2:

In your comments to section “5. Review - Comments to the Author” below you write:

“*Experiments, statistics, and other analyses are performed to a high technical standard and are described in sufficient detail*.”

We therefore believe that the “negative” response here might be a mistake. Nevertheless, we will briefly describe the reasoning of our analysis to highlight why we believe that our analyses were appropriate to answer the research question.

We aimed to model the main variable (exercise heart rate) with a within-subject linear mixed model that was built to address the main research question of interest, i.e., determine the overall mean short-term response in exercise heart rate, while accounting for potential long-term effects, as well as intra-individual and inter-individual variability (Goltz et al., 2018, Senn et al., 2011).

The secondary descriptive analyses were performed in accordance with the study design by performing intra-individual comparisons between repeated measurements at recovered versus strained states in a similar fashion as recommended for single-subject research designs (Barker et al., 2011). Therefore, the descriptive analyses served to verify intra-individual training strain responses within the given study sample. Training stain responses of at least *moderate* individual effect sizes were observed in every athlete for a minimum of four different monitoring measures (i.e., creatine kinase and perceived recovery and stress ratings; for reference please see the Supporting Information file “*S1 Table. Summary of individual monitoring data*”). Then, individual effects were summarized for descriptive purposes. On average, *moderate* to *large* individual effect sizes were observed for creatine kinase and perceived recovery and stress ratings (*Table 1*, *Figure 3*). These findings support the appropriateness of the implemented study design for describing different levels of recovery and training strain during repeated microcycles in every athlete. As substantial training strain related responses in creatine kinase and perceived recovery and stress ratings were observed in every athlete, we are confident that the Mondays and Fridays measurements actually represented contrasting states for every athlete in our study within the observational period which supports internal validity of the study design.

References:

- Barker J, McCarthy P, Jones M, Moran A. Single case research methods in sport and exercise psychology. Abingdon England, New York: Routledge; 2011.
- Goltz FR, Thackray AE, King JA, Dorling JL, Atkinson G, Stensel DJ. Interindividual Responses of Appetite to Acute Exercise: A Replicated Crossover Study. Med Sci Sports Exerc. 2018; 50: 758–768. <https://doi.org/10.1249/MSS.0000000000001504>
- Senn S, Rolfe K, Julious SA. Investigating variability in patient response to treatment--a case study from a replicate cross-over study. Stat Methods Med Res. 2011; 20: 657–666. <https://doi.org/10.1177/0962280210379174>

# Question 3

Have the authors made all data underlying the findings in their manuscript fully available?

The [PLOS Data policy](http://www.plosone.org/static/policies.action#sharing) requires authors to make all data underlying the findings described in their manuscript fully available without restriction, with rare exception (please refer to the Data Availability Statement in the manuscript PDF file). The data should be provided as part of the manuscript or its supporting information, or deposited to a public repository. For example, in addition to summary statistics, the data points behind means, medians and variance measures should be available. If there are restrictions on publicly sharing data—e.g. participant privacy or use of data from a third party—those must be specified.

- **Reviewer #1: No**
- Authors’ response to questions 3:

Maye there had been technical issues, so that the reviewer was not able to access the full material that was uploaded with the manuscript. I have recently double checked our submission, and everything was properly uploaded. Please contact the Academic Editor or the editorial office, if you do not have access to all material and links as uploaded and listed below:

The main dataset was uploaded as Supporting Information file “*S1 Dataset. Original data*” (S1_dataset.xlsx) during submission.

In addition, and as referenced in the data availability statement, supplementary material availability statement (lines 653-657) and in the main text (lines 244-245), a full data and analysis documentation was deposited at the Open Science Framework at <https://doi.org/10.17605/osf.io/up4ht>. Therefore, each single data and analysis file from our study was made available upon submission, which allows full reproducibility of all analyses, results, tables and figures (exceptions: “*Fig 1. study design*” and “*Fig 2. Submaximal shuttle-run test*”).

# Question 4

Is the manuscript presented in an intelligible fashion and written in standard English?

PLOS ONE does not copyedit accepted manuscripts, so the language in submitted articles must be clear, correct, and unambiguous. Any typographical or grammatical errors should be corrected at revision, so please note any specific errors here.

- **Reviewer #1: Yes**
- Authors’ response to question 4: No comment

# Question 5

Review Comments to the Author

Please use the space provided to explain your answers to the questions above. You may also include additional comments for the author, including concerns about dual publication, research ethics, or publication ethics. (Please upload your review as an attachment if it exceeds 20,000 characters)

**Reviewer #1:** Review of the manuscript PONE-D-20-22225, entitled "Monitoring training and recovery responses with heart rate measures during standardized warm-up in elite badminton players":

**The article presents a study to evaluate To investigate short-term training and recovery-related effects on heart rate during a standardized submaximal running test.
The study presents the results of original research.**

- **Reviewer #1 comment:**

**The article don’t adheres to appropriate reporting guidelines and community standards for data availability as, results reported and the entire draft have been already published in other on this site:**
[**https://www.researchgate.net/publication/343141060_Monitoring_training_and_recovery_responses_with_heart_rate_measures_during_standardized_warm-up_in_elite_badminton_players**](https://www.researchgate.net/publication/343141060_Monitoring_training_and_recovery_responses_with_heart_rate_measures_during_standardized_warm-up_in_elite_badminton_players)

- Authors’ response:

We purposefully decided to publish the preprint and its underlying data specifically in accordance to and because of the PLOS submission guidelines and policies.

As stated in the submission guidelines, PLOS and its associated journals clearly “*encourages authors to post preprints as a way to accelerate the dissemination of research*” (for reference: <https://journals.plos.org/plosone/s/preprints>). This includes the publication of the full manuscript in the pre-submission version, and all its accompanying results, material, and data. The open and findable dissemination of the manuscript and supporting material on repositories like SportRxiv or ResearchGate prior to submission and prior to peer-review is an essential component of preprints and open science. Based these PLOS guidelines we decided to publish a preprint to adhere to open science practices.

In addition, PLOS also “*require[s] authors to make all data necessary to replicate their study’s findings publicly available without restriction*” (<https://journals.plos.org/plosone/s/data-availability>). As mentioned above, this was also done in accordance to PLOS data sharing policy and using the Open Science Framework as one of the PLOS recommended repositories (<https://journals.plos.org/plosone/s/recommended-repositories>).

Finally, we followed the PLOS statistical reporting guidelines as outlined by Lang and Altman (2013).

Reference:

- Lang, T. A., & Altman, D. G. (2013). Basic statistical reporting for articles published in clinical medical journals: The SAMPL Guidelines. In P. Smart, H. Maisonneuve, & A. Polderman (Eds.), Science editors' handbook (2nd ed., pp. 175–182). EASE (European Association of Science Editors).
- **Reviewer #1 comment:**

**Experiments, statistics, and other analyses are performed to a high technical standard and are described in sufficient detail.**

**Conclusions are presented in an appropriate fashion. Nevertheless, not all are supported by the data.**

- Authors’ response:

We will try to address all concerns raised by reviewer with respective responses to the comments listed below.

- **Reviewer #1:**

**The article is presented in an intelligible fashion and is written in standard English**

**In my opinion, specific issues must to be amended and considered before acceptance.**

- **Reviewer #1, comment #1:
  The authors state to investigates ten elite badminton players, 7 females and 3 male. As is well known, how the serum concentration of creatine kinase and urea can change depending on the gender (in a example, previous studies reported that the females presented with a higher CPK peak and a greater relative increase in serum CPK levels after 50 maximal eccentric contractions of the arm flexor muscles, despite significantly lower baseline levels compared to males). Is missed to present the results without including males in the presented means.**
- Authors’ response to comment #1:

Thank you for highlighting this concern.

We agree and we are aware that males typically have higher baseline values of creatine kinase and urea. This aspect was considered when calculating individualized reference ranges for creatine kinase and urea as part of the Supplementary Information material “*S1 Appendix. Individual Player Reports*”. Here, gender specific prior distributions were used to perform the individualization procedure for calculating individual reference ranges. We also agree that responses to exercise may differ between gender.

We had previously avoided gender specific sub-group analyses or presentation of separate results for gender for the following reasons:

1. The concern raised by the reviewer of gender-related effects are related to the blood-borne measures creatine kinase and urea. Our main analysis for the non-heart rate variables was the calculation of intra-individual effect sizes for the mean difference between recovered and strained states. Hence, we were interested if substantial differences between measurements at the two contrasting time points were observable at the intra-individual level and for every player.

When comparing the intra-individual effect sizes, there was no gender-related effect visible which indicates that neither females nor males tends to show higher/lower responses. Please see the Figures A and B on the next page (rebuttal letter, page 8) on why we have no reasons to believe that the effects of interest were affected by gender in our study sample.

For reasons of clarity, we therefore initially decided to only present pooled analyses and summary statistics. Nonheart rate related variables were only used to describe training strain related short-term effects, and therefore to describe the appropriateness of our study design to compare contrasting time points during repeated microcycles in our study sample.

The separate presentation of summary statistics split by gender would go to substantial costs of clarity, while not adding any substantive information in the specific case of our study sample.

2. As outlined in 1, intra-individual effects were not affected by gender. Yet, it was very important for us to be able to provide the dataset used for this study to allow full reproducibility of all our analyses and results. The main difficulty of publishing original data from elite athletes at national level, is that it is very difficult the successfully de-identify the original dataset and make sure that athletes are not identifiable in retrospect.

To ensure this, we avoided to publish any data that would make a potential identification of athletes more likely without being relevant for the main analyses. As described in 1., gender had no clear effect on the intra-individual effect sizes. In our study, only intra-individual effects were of interest (i.e.: Was there a substantial mean difference present between recovered and strained measurements for every athlete?). Neither the players individual mean values at recovered and strained state were of interest, nor the average mean values.

This why we did not provide further details on the individual players age, height, weight, gender and why we also did not provide any information on the time frame or the year the study was conducted. Any of this information would make it far more likely to be able to identify at least some of the athletes in retrospect. As mentioned above, the results and analysis as currently performed are not substantially affected by gender, and we would therefore like to not include information like gender or respective sub-group analyses in the manuscript or its accompanying material for reasons of ensuring the athletes privacy.

3. Sample size was too small to perform proper, formal sub-group analyses. Even excluding the three male players would reduce the respective sample size by 30%, which increases further uncertainty in the calculated statistics.
4. We had no reason to believe that responses in exercise heart rate would be clearly affected by gender. As exercise heart rate was the main variable of interest, we decided to only perform pooled analyses to keep analyses consistent for all variables.

Fig A: Distribution and histograms of individual effect sizes for comparison between recovered and strained state. Variables: creatine kinase (natural logarithm), urea (natural logarithm), perceived recovery and stress ratings (PPC, OR, MS, OS), Countermovement jump, jump efficiency (multiple rebound jump), exercise heart rate, rating of perceived exertion. Red: female (f), green: male (m).

Fig B: Individual effect sizes for comparison between recovered and strained state. Variables: creatine kinase (natural logarithm), urea (natural logarithm), perceived recovery and stress ratings (PPC, OR, MS, OS), Countermovement jump, jump efficiency (multiple rebound jump), exercise heart rate, rating of perceived exertion. Red: female (f), green: male (m).

5. Additional gender-specific analyses would, in theory, mainly be relevant for blood-borne measures, which were not of primary interest. Hence, performing additional sub-group analyses and providing respective tables would make the results and appendix even more extensive, without being directly relevant to the main findings.

To summarize:

As mentioned in the beginning of this response to comment #1, we fully agree that gender can in general be expected to affect average values and potentially exercise responses in creatine kinase and urea.

Nevertheless, there were no gender-related differences visible in any of the monitoring markers in our specific sample. For reasons of clarity, we therefore prefer to only present results of individual effect sizes on pooled data. We believe that additional sub-group analyses would further complicate the presentation of the results, while there is no additional information gained in our specific case. Moreover, the small sample size would not allow formal analyses of gender differences.

Revisions:

As a compromise between generally valid criticism of the reviewer (gender-related effects are typically relevant) and the circumstance of our specific study (no gender effects observable; substantially reduced sample size when only analyzing females; issues with maintaining de-identification), we added additional summary statistics for females only for creatine kinase and urea within the Table 1. Please see the revisions in Table 1, page 11: Mean ± SD for females only: CK Rec 120 ± 44 U/L, CK Strain 212 ± 92 U/L, Urea – Rec 26 ± 7 mg/dL, Strain 28 ± 6 mg/dL.

- **Reviewer #1, comment #2:**

**Moreover, is missed the race difference, if there are some, as higher levels of total serum creatine kinase activity have been reported in black compared with white.**

- Authors’ response to comment #2:

Thank you for highlighting this. However, there were no black athletes in our sample.

- **Reviewer #1, comment #3:
  In the other hand, other anthropometrics measurements are missed in the study to better understanding the results (the level of muscle mass and the lower limbs length as they can change the muscle efficiency to develop CMJ or the shuttle run test, and , therefore , can change the muscle damage and the changes in CPK after exercise or the aforementioned tests).**
- Authors’ response to comment #3:

Thank you for highlighting this aspect. We agree that additional measures could be potentially useful for better understanding the results.

Due to the observational character of the study we were not able not perform any additional measurements that allowed us to estimate, for example, players’ muscle mass or lower limb length. We were only able to collect data directly related for the evaluation of monitoring markers within the given study design. We therefore cannot provide data related to muscle mass or lower limb length.

In addition, for the reasons mentioned above to ensure that data will remain as de-identifiable as possible, we purposefully did not include additional information that was not directly related to the main analysis of exercise heart rate.

Revision:

We included a respective statement to in the “Limitations and strengths of the study” section (lines 469-471): “Finally, it might also be valuable to evaluate the effects of potential influencing factors (e.g. anthropometrics, body composition) to better understand inter-individual differences in training and recovery responses.”

- **Reviewer #1, comment #4 Line 264:
  The authors state “including 4–14 measurements per player”, Were not the same measurements for all players?**
- Authors’ response to comment #4:

The reviewer is correct. Due to the reasons mentioned in the limitations section (lines 429-436), we were not able to collect the maximum amount of data for every player. These circumstances are common and unfortunately unavoidable in longitudinal field studies with elite athletes.

As discussed in lines 429-436, this clearly represents a limitation of the study. We did our best to address this limitation by respective analytical decisions and methods:

Sub-optimal data structure will unavoidably influence inferential statistics and results while probably increasing uncertainty in the estimates. We therefore performed linear mixed models which, in contrast to “classical repeated measures analysis”, generally allow data to be used for analysis even in presence of missing data and they “naturally handle uneven spacing of repeated measurements” (Seltman, 2018), as was the case in our study.

In addition, the within-subject standard deviations, which were used for calculating individual effect sizes, were calculated by polling intra-individual variability by accounting for different number of observations per athlete. In the case of exercise heart rate, this simple estimate of within-subject variability for recovered measurements was quite similar to the within-subject variability estimate from the linear mixed model. In total, 95 exercise heart rate measurements were included in the linear mixed model (line 264).

Finally, individual exercise heart rate time series (*Figure 4*) as well as full individual player reports (*“S1 Appendix. Individual Player Reports”*) were provided for every player to transparently allow the reader to visually evaluate the effects summarized by the inferential statistics.

Reference:

- Chapter 15 Mixed models: Seltman, H. J. (2018). Experimental Design and Analysis. <http://www.stat.cmu.edu/~hseltman/309/Book/Book.pdf>
- **Reviewer #1, comment #5 Line 302:
  The authors state “Our findings are consistent with previous studies that reported increased levels of CPK 12 hours after badminton-specific training” nevertheless this study was performed on six national male badminton players, who can increase much more the CPK levels in comparison to females.**
- Authors’ response to comment #5:

As mentioned above, we agree with the reviewer that males typically show higher average creatine kinase values and that responses may differ between females and males.

However, we think that our statement in lines 301-302 is neither in conflict with this fact, nor that it is thereby incorrect. Majumdar et al. (1997) found increased levels of creatine kinase following badminton-specific training. In our study, we also observed increased levels of creatine kinase following mainly badminton-specific training. Although there are typically gender-related differences expectable, both studies reported increased levels of creatine kinase following badminton training and can therefore be considered consistent in general terms: Badminton-specific training may elicit higher creatine kinase values post exercise. Increased levels of creatine kinase following badminton-specific training was observed both in males [acute (Majumdar et al., 1997) and short-term (Barth et al., 2019)] and in females [short-term (our findings)]. Therefore, we consider our creatine kinase results consistent with the referenced articles, despite the differences in gender.

In contrast to the reviewer’s concern, we believe that our statement is especially adequate because there were differences in gender between studies. When males typically show higher creatine kinase values (and responses), one would not necessarily expect similar effects in females. However, as we observed changes in similar direction, despite studying mainly female athletes, the responses observed in our study are indeed consistent with the observations in males. Please note that with our analytical approach, the magnitude of response was compared to the variability in measurements. Therefore, even small but consistent responses would be identified as “clear” response.

References:

- Barth, V., Käsbauer, H., Ferrauti, A., Kellmann, M., Pfeiffer, M., Hecksteden, A., & Meyer, T. (2019). Individualized Monitoring of Muscle Recovery in Elite Badminton. Frontiers in Physiology, 10, 778. <https://doi.org/10.3389/fphys.2019.00778>
- Majumdar, P., Khanna, G. L., Malik, V., Sachdeva, S., Arif, M., & Mandal, M. (1997). Physiological analysis to quantify training load in badminton. British Journal of Sports Medicine, 31(4), 342–345. <https://doi.org/10.1136/bjsm.31.4.342>
- **Reviewer #1, comment #6 Line 303-304:
  The authors state “As well as in response to repeated weekly microcycles in endurance athletes [10] and badminton players [11]”, developed in non-comparable 14 elite junior swimmers and triathletes and 17 elite male badminton players.**
- Authors’ comment to comment #6 303-304:

We again agree that studied subjects were different between the referenced studies and our study.

In the following, we will briefly outline why we consider these references adequate for the respective statement:

In our study, we compared two contrasting time points during repeated preparatory training cycles. This study design required that the two time points represent contrasting states (i.e., ‘recovered’ versus ‘strained’ state). This important aspect was communicated with the coaches prior to the study period, and only measurements were included that satisfied the requirement of recovered or strained time points based on the training schedule. For example, measurements were not considered for analyses if training or competition schedules conflicted with the inclusion criteria (please see lines 431-434). More importantly, to verify that the two time points represent contrasting states, the different monitoring markers were required to display respective short-term effects.

To make sure that we would be potentially able to observe differences in the various monitoring markers in the ‘recovered’ and ‘strained’ time points, we used a study design that has previously been shown to display different levels of creatine kinase and perceived recovery and stress ratings (lines 421-424). As the reviewer correctly mentioned, the athletes in the two previous studies that have implemented the same study design were different compared to our sample (Barth et al., 2019; Hecksteden et al., 2017). Therefore, we could not be entirely sure that their findings would translate to our study sample and our training context. However, our findings were consistent with those from the referenced two studies, as increased levels of creatine kinase were observed in all three studies, despite differences in the subjects and differences in sport when compared to the Hecksteden et al. (2017) study. We therefore believe that our statement “*Our findings are consistent with previous studies …*” is therefore appropriate.

References:

- Barth V, Käsbauer H, Ferrauti A, Kellmann M, Pfeiffer M, Hecksteden A, et al. Individualized Monitoring of Muscle Recovery in Elite Badminton. Front Physiol. 2019; 10: 778. <https://doi.org/10.3389/fphys.2019.00778>
- Hecksteden A, Pitsch W, Julian R, Pfeiffer M, Kellmann M, Ferrauti A, et al. A new method to individualize monitoring of muscle recovery in athletes. Int J Sports Physiol Perform. 2017; 12: 1137–1142. <https://doi.org/10.1123/ijspp.2016-0120>
- **Reviewer #1, comment #6 Line 475-478**:
  **The authors state “since the HRex response is known to be influenced by many factors, practitioners should consider the potentially overlapping effects of acute and short-term training load changes, long-term training adaptations, and external confounding factors when interpreting HRex.” I agree, and this is the reason why I think that many of the conclusions can’t be stated for this study.**
- Authors’ comment to comment #6 475-478:

Please also see our responses to your specific comment above.

As outlined in the statement in lines 475-478, we believe that heart rate measure in sports practice can be potentially influenced by many factors. Therefore, we included this statement in the conclusions, which should, from our point of view, be considered when trying to generalize our findings beyond our specific study model. This concluding statement was aimed to caution against too optimistic generalization from our specific study findings. Nevertheless, as outlined in the following, in our study we aimed to account for as many potential influencing factors as possible with several methodological and analytical decisions. Furthermore, we aimed to frame and discuss our findings in a reserved manner to avoid overstatements of our findings.

Tests were always performed at the same time of the day for all players, which eliminates potential effects due to circadian rhythm (please see lines 114-115 and Fig 1.). Furthermore, temperature was recorded for every session. Observed intra-individual difference between recovered and strained tome points were deemed neglectable compared effects of heat and cold in the previous literature (see lines 385-389). Since tests were performed in the morning sessions as the first part of the (pre-) warm-up routine, immediate effects of previous exercise (like immediate or acute exercise-related fatigue) could also be excluded.

Based on the repeated measures design and our statistical analyses, we aimed to determine short-term effects while controlling for (linear) long-term changes over the study period (lines 210-213 and 219-221). We further accounted for inter-individual differences in HRex or inter-individual differences in short-term or long-term effects by specifying respective random effects. We therefore believe that a substantial number of important (potential) influencing factors were controlled or sufficiently accounted for with our study design and statistical analysis.

# Question 6

Do you want your identity to be public for this peer review?

- **Reviewer #1: Yes: PhD. Monica Solana-Tramunt**
- Authors’ response to question 6:

Thank you for supporting open peer review. We believe that open peer review is very important step forward towards a modern and open science culture.
